# A Hierarchical Routing Graph for Supporting Mobile Devices in Industrial Wireless Sensor Networks

**DOI:** 10.3390/s21020458

**Published:** 2021-01-11

**Authors:** Sangdae Kim, Cheonyong Kim, Hyunchong Cho, Kwansoo Jung

**Affiliations:** 1 Division of Computer Science and Engineering, Kongju National University, Cheonan 31080, Korea; sdkim.cse@gmail.com; 2 National Institute of Supercomputing and Networking Advanced KREONET Center, Korea Institute of Science and Technology Information, Daejeon 34141, Korea; cykim0807@kisti.re.kr; 3 Research Institute for Computer and Information Communication, Chungbuk National University, Cheongju 28644, Korea; hccho@chungbuk.ac.kr; 4 Department of Fintech, Daejeon University, Daejeon 34519, Korea

**Keywords:** Industrial Wireless Sensor Networks (IWSNs), mobility support, graph construction, real-time, reliability

## Abstract

As many industrial applications require real-time and reliability communication, a variety of routing graph construction schemes were proposed to satisfy the requirements in Industrial Wireless Sensor Networks (IWSNs). Each device transmits packet through a route which is designated based on the graph. However, as existing studies consider a network consists of static devices only, they cannot cope with the network changes by movement of mobile devices considered important in the recent industrial environment. Thus, the communication requirements cannot be guaranteed because the existing path is broken by the varying network topology. The communication failure could cause critical problems such as malfunctioning equipment. The problem is caused repeatedly by continuous movement of mobile devices, even if a new graph is reconstructed for responding the changed topology. To support mobile devices exploited in various industrial environments, we propose a Hierarchical Routing Graph Construction (HRGC). The HRGC is consisted of two phases for hierarchical graph construction: In first phase, a robust graph called skeleton graph consisting only of static devices is constructed. The skeleton graph is not affected by network topology changes and does not suffer from packet loss. In second phase, the mobile devices are grafted into the skeleton graph for seamless communication. Through the grafting process, the routes are established in advance for mobile device to communicate with nearby static devices in anywhere. The simulation results show that the packet delivery ratio is improved when the graph is constructed through the HRGC.

## 1. Introduction

Industrial networks are exploited for various industrial fields such as inventory system [1], monitoring system [2,3], and control system [4,5] based on collected information in order to reduce the cost and improve the productivity [6,7]. For example, in the case of the inventory system [1], it could prevent the overload or shortage condition of the product and the raw materials through the detection of them. In the case of the monitoring system [2,3], it could predict the failure of the equipment in advance and prevent the quality and productivity degradation from the equipment failures by it constantly monitors currently operating equipment. Furthermore, the control system [4,5] could effect cost reductions through the process automation based on the collected information. The safety system [8,9] could minimize the damage through the immediate action about the various disaster. These systems require information that is collected in a timely manner with high reliability in order to attain efficiency, productivity, automation, etc. That is, the real-time, high-reliability packet transmission is key requirement of today’s industrial network [3,8,10].

To exploit the industrial network in various industrial fields, studies have been developed from the wired to the wireless communication environments. The wired networks such as HART [11] and FieldBUS [12] are the simplest and most reliable network. However, there exists difficulty in the installation and maintenance of the wire and installing and protect the wire is expensive. To alleviate the drawbacks of the wired network, wireless networks such as ZigBee [13], WIA-PA [14], ISA 100.11A [15], WirelessHART [16], etc. have become popular. Wireless networks can be installed more easily than wired networks, and they also have the advantages of low cost and that protection for wiring is not required. Especially, the wireless networks are essential to support the flexibility of the facilities that are required in today’s flexible industrial environments such as low-volume, high-variety production [17,18].

The wireless networks are originated from Wireless Sensor Networks (WSNs) exploiting the ZigBee [13]. However, WSNs have a difficult-to-satisfy low-delay, high-reliable requirement of various industrial systems because of the wireless collision and interference. Thus, the Industrial Wireless Sensor Networks (IWSNs) represented by WirelessHART have been studied in order to satisfy the requirements of the industrial environment. The network management device called Network Manager (NM) of WirelessHART constructs a directed graph about the whole network topology based on the collected information of each field device. After graph construction, NM calculates the channel and time slot exploited to transmit packet and commands to other devices. Next, NM notifies the results of the calculation to every field device in the network. It could prevent the problem of the wireless collision and interference. Thus, the IWSNs would facilitate the low-delay, high-reliable packet transmission.

However, as the existing IWSNs focus on alleviating the problem of wired systems, such as wire installation and maintenance cost, they consider a network consisting of static devices only. Therefore, existing studies cannot adequately respond to network changes that occur when mobile devices are exploited. As mentioned earlier, the existing scheme determines the route of each device for communication based on the network topology for low-delay and high-reliability. However, the movement of mobile device causes network topology changes, and it requires recalculating of routes. As a result, each device cannot transmit packet through a route assigned by NM, and the packet loss causes critical problems such as malfunctioning equipment, production process delay in various industrial applications. Furthermore, even if NM performs recalculation of routes about changed network topology, the problem is not solved because the network topology changes constantly due to the movement of the mobile devices.

To prevent route failure caused by movement of mobile devices, we propose a Hierarchical Routing Graph Construction (HRGC). The main idea of the HRGC is to divide the static and mobile device to construct a graph. The HRGC is largely consisted of two phases to construct hierarchical routing graphs: (1) constructing a skeleton graph and (2) grafting mobile devices into the skeleton graph. First, a steady routing graph called skeleton graph consisting only of static devices is constructed. As the graph represents a connection between static devices, it is not affected by network topology changes due to the movement of mobile devices. Thus, the construction of the skeleton graph could ensure the communication route of the static devices. Second, the mobile devices are grafted into the skeleton graph for seamless communication between the static devices and mobile devices. As the movement of mobile device is unpredictable and continuous, it constructs the virtual routes in advance for communication with the mobile device in the static devices which are member of the skeleton graph. That is, as the static device constructs a route on the assumption that there is mobile device near it, the mobile device could transmit packets anywhere through a nearby static device on the skeleton graph.

The remainder of this paper is organized as follows. In Section 2, we briefly introduce requirements of routing graph construction in WirelessHART standard first. In addition, we describe the representative graph construction schemes and studies for reducing the time to detect network changes. In Section 3, we explain a graph construction scheme that minimizes the network changes to improve the packet delivery ratio. The performance evaluation results by simulation are provided in Section 4. Finally, the HRGC and performance evaluation results are summarized in Section 5.

## 2. Related Work

In this section, we describe reviews of the existing routing graph construction scheme in Section 2.1 and indicate the problem of existing routing graph construction scheme in Section 2.2.

### 2.1. Routing Graph Construction Scheme

The graph construction is one of important functions in WirelessHART. However, the WirelessHART standard [16] does not provide specific routing scheme, but the requirements are described. Thus, various studies have been proposed to construct graph which satisfy the requirements. In this section, we briefly summarize the existing graph construction schemes.

Han et al., in  [19], proposed an algorithm to satisfy the low delay and high reliability required in IWSNs. Han et al. [19] exploits the (k,m)-reliability concept to ensure the reliability. *k* and *m* represent the number of ingoing and outgoing edges of each network device, respectively. When the network manager constructs the graph, each device would have at least two ingoing and outgoing edges. In order words, each device has a primary and alternative path in order to respond to the case where one path is broken. In addition, to achieve real-time transmission, a device with minimum hop counts from itself to the gateway is firstly included in the graph.

Zhang et al., in  [20], proposed constructing a graph for energy-balanced routing to maximizing network lifetime. Zhang et al. [20] also construct a reliable routing graph by creating two paths from each device to the gateway. To construct the energy-balanced graph, they proposed a joint routing for maximizing network lifetime (JRMNL) algorithm. The network manager collects the information about a device communication load, residual energy, and link transmission power and calculates link cost. Each device would choose the optimal next hop by comparing the link cost of all the possible links.

Li et al., in [21], exploit an artificial bee colony (ABC) to construct a minimum spanning tree (MST). The proposed scheme cannot find an optimal tree; however, it could find multiple solutions in conformity with requirements. In the initialization phase, the ABC randomly generates initial populations containing food sources. The employed bee compares the new food source with the original food source and selects a solution with high fitness as a candidate solution. When the searching work is finished, the employed bees share the food source information with the onlooker bees. The onlooker bees choose the food source according to probability. The higher the food source’s fitness value, the greater the probability of being selected. Finally, the scout bees search for a new food source. The above process would be repeated for the specified maximum number of iterations. If the maximum number of iterations is reached, the final best food source becomes the global solution for constructing MST.

### 2.2. Problem of Existing Graph Construction Scheme

In this section, we explain a problem on the graph caused by the movement of the mobile device. As mentioned in the introduction, as the existing WirelessHART standard is designed without the consideration of the mobile devices, the movement of the mobile device causes the network changes. Finally, this phenomenon leads to reduced performance.

**Figure 1:**
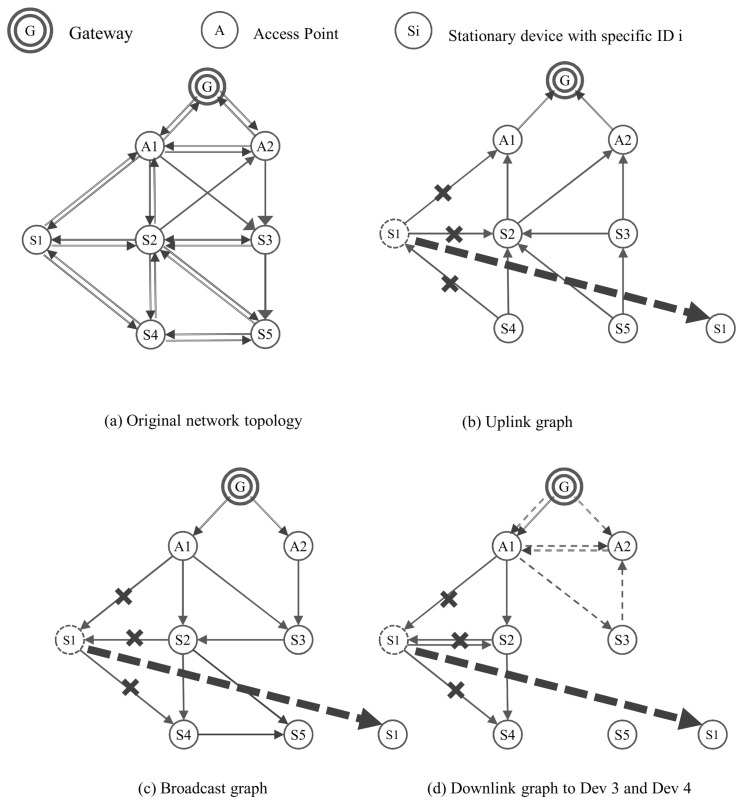
An example of network changes problem on the constructed graphs by the authors of [19].

To explain a network changes in more detail, we exploit the relatively simple graph construction scheme [19] as an example. Figure 1 shows an example of upstream/downstream graph based on the construction scheme and the phenomenon according to the network changes. Based on the original topology graph (Figure 1a), the network manager constructs the uplink graph (Figure 1b) with two outgoing edges at the devices for receiving data from the device and the broadcast graph (Figure 1c) with two ingoing edges at the devices to broadcast a command to all devices. Then, to manage each device separately, the  network manager generates the downlink graph for each device. In the case of Figure 1d, it shows the downlink graph of device S3 (thin dotted line) and S4 (thin solid line).

In the graphs constructed earlier, we assume that the device 1 is a mobile device and shows the problem that occurs when device 1 moves to the location of device 5. Device 1 loses the paths of its uplink, downlink, and broadcast graph. Device 2, device 4, and access point A1 also lose the path associated with the device 1. These paths would be recovered when the mobile device is discovered through the neighbor discovery process [19,22] or the mobile device rejoin to the network. That is, device 1 cannot communicate with the gateway before path recovery through the discovery process or rejoin process. In addition, as the graph of the some static devices related the device 1 is disconnected, it has a bad influence on the real-time and high-reliable communication of the some static device. Figure 1 shows that one mobile device exists in a relatively small network, which may seem to have a small impact. However, the impact of multiple mobile devices moving in a large network is adversely affected in many aspects as seen in performance evaluations.

To summarize the problem with network changes caused by the movement of mobile device, the movement of the mobile device would cause (1) path failure of some static device and (2) path disconnection problem of the mobile devices. Both phenomena cause a critical problem in IWSNs that require low-delay and high-reliability. Especially, the problem persists until the network manager realizes the network change and reflects it. In order to solve this problem, methods for quickly detecting network changes have been proposed [22], but the limitations exist to reflecting the continuous network change caused by mobile devices. Therefore, we conclude that network changes due to the mobile device should be minimized, and describe the scheme to minimize the changes in Section 3.

## 3. Hierarchical Routing Graph Construction for Supporting Mobile Devices

In this section, we describe Hierarchical Routing Graph Construction (HRGC) for supporting mobile devices by solving the problem caused by the movement of mobile devices. The HRGC consists of two main parts: (1) a skeleton graph consisting of only static devices and (2) mobile devices that are grafted into the skeleton graph. The skeleton graph construction could prevent the path failure problem of the static devices, and the grafting process could be enabled to communicate between the skeleton graph and mobile devices.

This section is divided into three parts. In Section 3.1, we summarize the notations to be used throughout this paper. In Section 3.2, we explain the skeleton graph construction scheme for preventing the network changes from the mobile device. In Section 3.3, we describe how to graft the mobile devices into the constructed skeleton graph. As our main idea is the distinction between static devices and mobile devices, the idea could be applied to various graph construction scheme. In this section, we apply our ideas to [19] to help understand the HRGC.

### 3.1. Notations

In this section, we explain the notations to be used throughout this paper. *g* denotes the gateway. G(V,E) means the original network topology. $G_{B}\left(V_{B},E_{B}\right)$, $G_{U}\left(V_{U},E_{U}\right)$, and $G^{R}\left(V^{R},E^{R}\right)$ denote the broadcast graph, uplink graph, and reversed graph about the original network topology G(V,E), respectively. *V* and *E* denote the set of devices and edges, respectively. $V_{AP}$ is set of the access point. $V_{B}$ and $E_{B}$ mean the set of devices and edges that compose the broadcast graph, respectively. Similarly, $V_{U}$ and $E_{U}$ mean the set of devices and edges that compose the uplink graph, respectively. $V_{static}$ means the set of static devices, and $V-V_{static}$ indicates the set of mobile devices.

### 3.2. Skeleton Graph Construction

**Figure 2:**
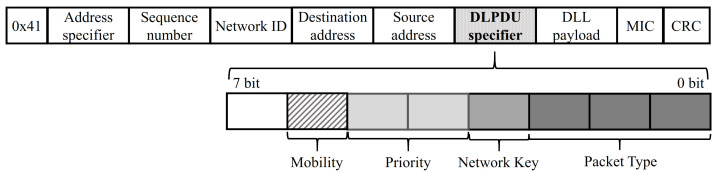
A modified data link protocol data unit (DLPDU).

In the existing WirelessHART standard, all devices are participated by exploiting the join process. We modify the join message to distinguish between the static and mobile device. Figure 2 shows the 7-bit Data Link Protocol Data Unit (DLPDU) specifier used in the standard. The specifier determines the message type, priority, etc. We exploit bit-6 in order to distinguish between static and mobile devices. When a device sends the join message to participate WirelessHART network, the static device sets a bit-6 as “0” and the mobile device sets a bit-6 as “1”. Through the modified join message, the network manager could determine the mobility of each device and reflect it in the graph construction process.

Algorithm 1 shows the algorithm to construct the broadcast graph consisting of static devices only. The network manager can classify a set of static devices by modified DLPDU when the device joins the network. The network manager constructs a skeleton graph, which is not affected by the mobile device, consisted of the static devices. As a connection between each static device and the gateway is constructed irrespective of the mobile device, the connection does not be damaged from the movement of the mobile devices. After broadcast graph including all of the static devices is constructed through the above-mentioned process, Algorithm 1 would be terminated and return the broadcast graph $V_{B}$.
**Algorithm 1 **Pseudocode for Skeleton Broadcast Graph Construction1:*G(V,E)* is the original graph.2:Initially $V_{B}=g\cup V_{AP}$ and $E_{B}$ contains all links from *g* to $V_{AP}$ ⊳ *g* is gateway, $V_{AP}$ is set of APs3:**while**$V_{B}\neq V_{static}$**do**4:    Find $S^{\prime }\subseteq V_{static}-V_{B}:\forall v\in S^{\prime },v$ has at least two edges from $V_{B}$ and no mobility.5:    **if** $S^{\prime }\neq \emptyset$ **then**6:        **for** $\forall v\in S^{\prime }$ **do**7:           Sort edges $e_{u,v}$ from $V_{B}$ according to ${\bar{h}}_{u}$          ⊳ *u* is one of neighbor nodes of *v*8:           Choose the first two edges $e_{u_{1},v}$ and $e_{u_{2},v}$9:           ${\bar{h}}_{v}$ = (${\bar{h}}_{u_{1}}+{\bar{h}}_{u_{2}}$)/2 + 110:        **end for**11:        Choose the device *v* with min ${\bar{h}}_{v}$12:        Add *v* to $V_{B}$ and add $e_{u_{1},v}$ and $e_{u_{2},v}$ to $E_{B}$13:    **else**14:        Find $S^{\prime \prime }\subseteq V_{static}-V_{B}:\forall v\in S^{\prime \prime },v$ has one edge $e_{u,v}$ from $V_{B}$ and no mobility.15:        **if** $S^{\prime \prime }\neq \emptyset$ **then**16:           **for** $\forall v\in S^{\prime \prime }$ **do**17:               ${\bar{h}}_{v}={\bar{h}}_{u}+1$18:               Calculate $n_{v}$, the # of its outgoing edges to $V-V_{B}$19:           **end for**20:           Choose the device *v* with maximum $n_{v}$, break tie using ${\bar{h}}_{v}$21:           Add *v* to $V_{B}$ and add $e_{u,v}$ to $E_{B}$22:        **end if**23:    **end if**24:**end while**25:**return**$G_{B}\left(V_{B},E_{B}\right)$

A more detailed description about Algorithm 1 follows. In this phase, the network manager considers only the static devices $V_{static}$ to construct a skeleton graph (line 4). To satisfy the (2,0)-reliability, the network manager chooses a device *v* that has at least two edges from the $V_{B}$ (line 5). If the candidates of *v* are more than two, the manager selects a device with lower average hop count *h* from the gateway (lines 8–10), and inserts the selected device *v* into the graph $V_{B}$ (line 13). Through this process, if the static device that has at least two edges are inserted to the graph $V_{B}$, the network manager inserts the remaining static device with one edges to the graph $V_{B}$ (lines 15–23). All static devices $V_{static}$ are connected $V_{B}$, the broadcast graph $G_{B}$ is returned, and the algorithm is terminated (line 26).

Algorithm 2 shows that the algorithm to construct the up-link graph with the HRGC. First, the network manager reverses the direction of the edges in the basic topology (line 3) and applies Algorithm 1 to the reversed graph (line 4). After constructing the broadcast graph of the reversed topology through this process, the network manager reverses the direction of the edges in the constructed broadcast graph (line 7) and returns the graph $G_{U}\left(V_{U},E_{U}\right)$ (line 9).
**Algorithm 2 **Pseudocode for Skeleton Uplink Graph Construction1:*G(V,E)* is the original graph.2:$G^{R}\left(V,E^{R}\right)$ is the reversed graph.3:Construct $G^{R}\left(V,E^{R}\right)$4:Construct $G_{B}\left(V_{B},E_{B}\right)$ from $G^{R}\left(V,E^{R}\right)$ by applying Algorithm 15:**if** $V_{B}=V_{static}$ **then**6:    $G_{U}\left(V_{U},E_{U}\right)={G_{B}}^{R}\left({V_{B}}^{R},{E_{B}}^{R}\right)$7:**end if**8:**return**$G_{U}\left(V_{U},E_{U}\right)$

**Figure 3:**
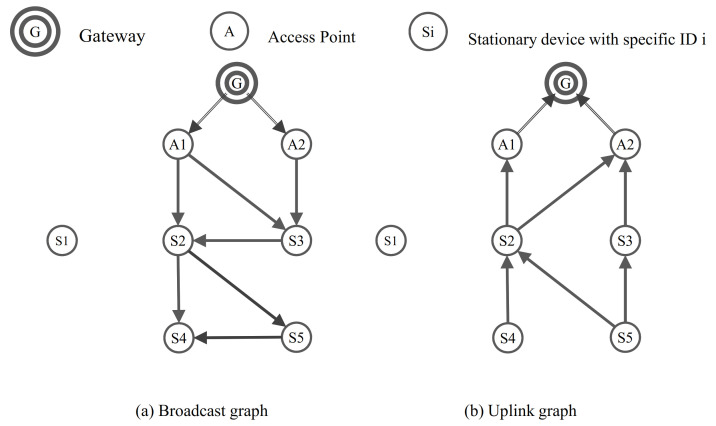
An example of skeleton graph construction by HRGC.

Figure 3 shows an example of skeleton graph construction that is applied with the HRGC to the original topology in Figure 1a. As shown in Figure 1, assume that the device 1 is a mobile device and other devices are the static device. When the device 1 moves to around the device 5, the paths of the static devices are not affected by the movement of a mobile device because the skeleton graph construction process produced except the mobile device. That is, the HRGC prevents the path failure of the static device by the movement of mobile device, it also avoids problems such as packet loss, network performance degradation.

### 3.3. Grafting Mobile Device into Skeleton Graph

In Section 3.2, we explained how to construct a skeleton graph to prevent path failure caused by the mobile devices. However, as the skeleton graph construction scheme considers only static devices, it does not solve the path disconnection problem of mobile devices. Therefore, in this section, we describe how to graft the mobile devices into a skeleton graph to prevent the path disconnection problem of the mobile devices.

As the communication path of mobile devices should constantly change according to the movement, the graph for communication of mobile devices cannot be constructed fixed such as the skeleton graph. That is, if a new graph is constructed each time according to network changes, packet omission of the mobile device would occur during the graph construction process. To solve the problem, we construct a graph for mobile devices on all static devices in advance. In other words, we construct a graph assuming that the mobile devices exist near all static devices.

Algorithm 3 shows the algorithm to graft the mobile devices into the skeleton broadcast graph $G_{B}$. First, the network manager distinguishes the set of mobile devices $S^{\prime }$ (line 4) and set of static devices $S^{\prime \prime }$ (line 5). If there is a mobile device that is not part of the broadcast graph, the network manager create a connection $e_{w,v}$ between the mobile devices *v* and all static devices *W* (line 9). All mobile devices *v* are connected $V_{B}$, the broadcast graph $G_{B}$ is returned, and the algorithm is terminated (line 14). Through grafting mobile devices into the skeleton broadcast graph, the mobile device could receive the broadcast message from gateway through the peripheral static device at any location.
**Algorithm 3 **Pseudocode for Grafting Mobile Device on to Skeleton Broadcast Graph1:$G_{B}\left(V_{B},E_{B}\right)$ is the skeleton broadcast graph.2:**while**$V_{B}\neq V$ **do**3:    Find $S^{\prime }\subseteq V-V_{B}:\forall v\in S^{\prime },v$ is mobile device4:    Find $S^{\prime \prime }\subseteq V_{B}:\forall w\in S^{\prime \prime },w$ is static device5:    **if** $S^{\prime }\neq \emptyset$ **then**6:        **for** $\forall v\in S^{\prime }$ **do**7:           **for** $\forall w\in S^{\prime \prime }$ **do**8:               Add *v* to $V_{B}$ and add $e_{w,v}$ to $E_{B}$9:           **end for**10:        **end for**11:    **end if**12:**end while**13:**return** $G_{B}\left(V_{B},E_{B}\right)$


The process for grafting the mobile devices into the skeleton uplink graph is similar to the process of grafting the mobile devices into the skeleton broadcast graph. Algorithm 4 shows the algorithm to graft the mobile devices into the skeleton uplink graph $G_{B}$. First, the network manager distinguishes the set of mobile devices $S^{\prime }$ (line 4) and set of static devices $S^{\prime \prime }$ (line 5). If there is a mobile device that is not part of the uplink graph, the network manager create a connection $e_{v,w}$ between the mobile devices *v* and all static devices *W* (line 9). All mobile devices *v* are connected $V_{U}$, the uplink graph $G_{U}$ is returned, and the algorithm is terminated (line 14). Through grafting mobile devices into the skeleton uplink graph, the mobile device could transmit the packet to gateway through the peripheral static device at any location.

**Figure 4:**
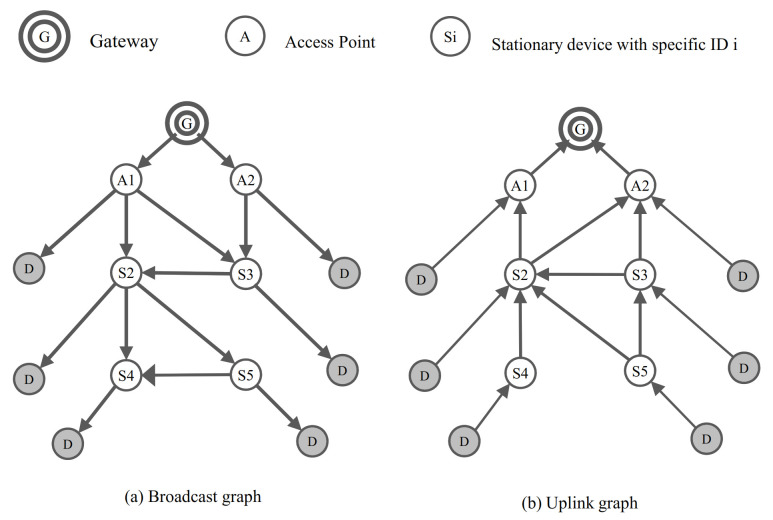
An example of grafting mobile device into skeleton graph.

Figure 4 shows an example of grafting mobile device *D* into skeleton graph in Figure 3. In Figure 4, the graphs were constructed assuming that the mobile device *D* was near all static devices. Based on the graph, the mobile device can receive the packet from the gateway wherever they are located and also can transmit the packet to the gateway. For example, the mobile device is located near the static device S4, the mobile device *D* can receive the broadcast message from the gateway through routes such as G → A1 → S2 → S4 → D. Likewise, if the mobile device *D* is located near the static device S4, the mobile device *D* can transmits its data packet to the gateway through routes such as S4 → S2 → A1 → G.
**Algorithm 4 **Pseudocode for Grafting Mobile Device on to Skeleton Uplink Graph1:$G_{U}\left(V_{U},E_{U}\right)$ is the skeleton uplink graph.2:**while**$V_{U}\neq V$**do**3:    Find $S^{\prime }\subseteq V-V_{U}:\forall v\in S^{\prime },v$ is mobile device4:    Find $S^{\prime \prime }\subseteq V_{U}:\forall w\in S^{\prime \prime },w$ is static device5:    **if** $S^{\prime }\neq \emptyset$ **then**6:        **for** $\forall v\in S^{\prime }$ **do**7:           **for** $\forall w\in S^{\prime \prime }$ **do**8:               Add *v* to $V_{U}$ and add $e_{v,w}$ to $E_{U}$9:           **end for**10:        **end for**11:    **end if**12:**end while**13:**return** $G_{U}\left(V_{U},E_{U}\right)$


## 4. Performance Evaluation

In this section, we evaluate the performance of the HRGC. To demonstrate the effectiveness of the HRGC, we compare the original Han [19] algorithm, the Han algorithm with NDP [22], and the Han algorithm with HRGC.

We have implemented the HRGC and other schemes in network simulator NS-3 [23]. In a 500 m × 500 m field, one-hundred devices are arranged in a lattice structure, and 1–20 devices are moving. The mobile devices move randomly according to the Random Way Point (The mobile devices move in different directions randomly in each simulation.) at a speed of 3 m/s, and it would be changed according to the performance evaluation factors. The transmission range of each device is 50 m, all devices transmit data to the gateway at every four seconds. In order to grasp the degradation of the transmission success ratio caused by network changes, the transmission of the wireless medium is assumed 100%. The total simulation time is 6400 s, and the simulation results are the average value of the results obtained 10 times for each simulation. The simulation factor and terms for performance evaluation are defined as follows.

- Connection Time is defined as the time that the mobile device could communicate with the network.
- Transmission Success Ratio is defined as the number of messages arriving at the gateway divided by the number of messages sent by the whole devices.
- Energy Consumption is defined as the average energy consumed by all devices for transmission, reception during the entire simulation time.
- Movement Speed indicates how fast the mobile device moves.
- The Number of Mobile Device is defined as the number of devices simultaneously moving in the network.

### 4.1. Connection Time Versus Movement Speed of Mobile Devices

**Figure 5:**
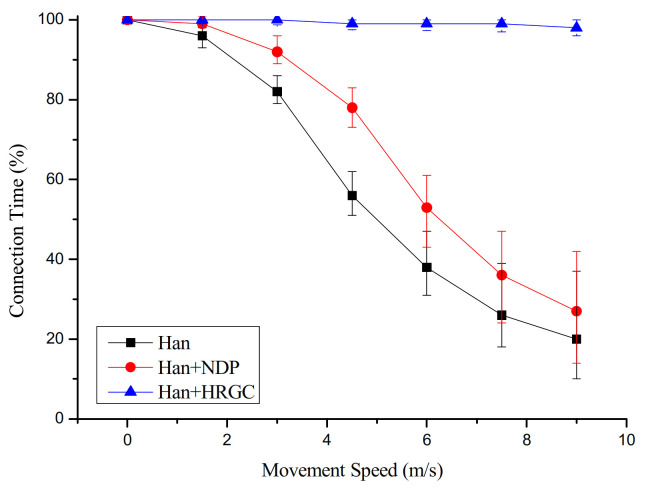
Connection time vs. movement speed of mobile devices.

Figure 5 shows the simulation result of the connection time versus movement speed of mobile device. In the case of the Han algorithm and Han algorithm with NDP, the connection time is degraded according to the increment of the movement speed of the mobile device. It is because the graph in these schemes is broken by the movement of the mobile device. That is, the connection between the mobile device and the network is disconnected until a new graph is constructed after detecting the network changes. Especially, if the movement speed is fast, the connection time is dramatically degraded because the mobile device moves to another location before the graph to respond to the network changes is applied.

However, as the Han algorithm with HRGC constructs the graph for communication with the mobile devices on all static devices, as shown Figure 4, the mobile device can communicate with the network anywhere.

### 4.2. Transmission Success Ratio versus Movement Speed and the Number of Mobile Devices

**Figure 6:**
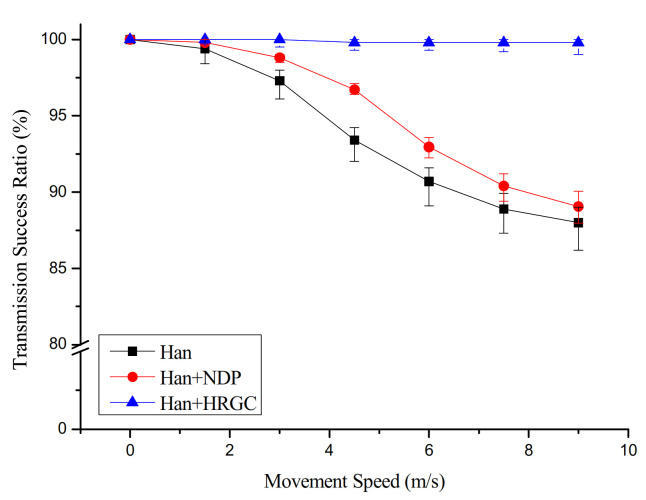
Transmission success ratio vs. movement speed of mobile devices.

Figure 6 shows the simulation result of the transmission success ratio versus movement speed of mobile device. In case of the Han algorithm and Han algorithm with NDP, the transmission success ratio is degraded when the mobile device moves faster. This phenomenon is not only directly related to connection time, but also caused by the path failure of the child device of mobile device. That is, the short connection time between the mobile device and the network causes the packet loss from the mobile device to the gateway. In addition, when the graph is constructed according to the above-mentioned schemes, the packet transmission success ratio is degraded due to the path failure between the mobile device and child devices by the movement of the mobile device. However, these problems are prevented in Han algorithm with HRGC because the algorithm constructs the graph for communication between the mobile device and the network in advance. That is, the skeleton graph consisting of the static device only does not be affected by the movement of the mobile device. Moreover, the mobile device could communicate with the network anytime, anywhere because of the mobile device grafting process.

**Figure 7:**
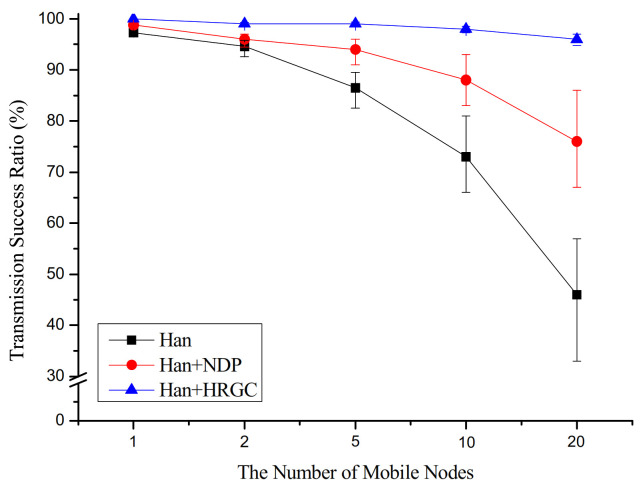
Transmission success ratio vs. the number of mobile devices.

Figure 7 shows the simulation result of the transmission success ratio versus the number of mobile devices. In case of Han algorithm and Han algorithm with NDP, the transmission success ratio is degraded according to the number of mobile devices. The increase in the number of mobile devices means an increase in the number of devices that cause network changes. That is, as the number of static devices associated with the mobile devices increases, the movement of the mobile devices will destroy the graph of a number of static devices. As shown in Figure 6, in the case of Han algorithm with HRGC, the skeleton graph is not affected by the movement of the mobile devices and the mobile devices could communicate with the network through the static devices around itself. Thus, the Han algorithm with HRGC shows highest packet transmission success ratio.

### 4.3. Energy Consumption versus Movement Speed and the Number of Mobile Devices

**Figure 8:**
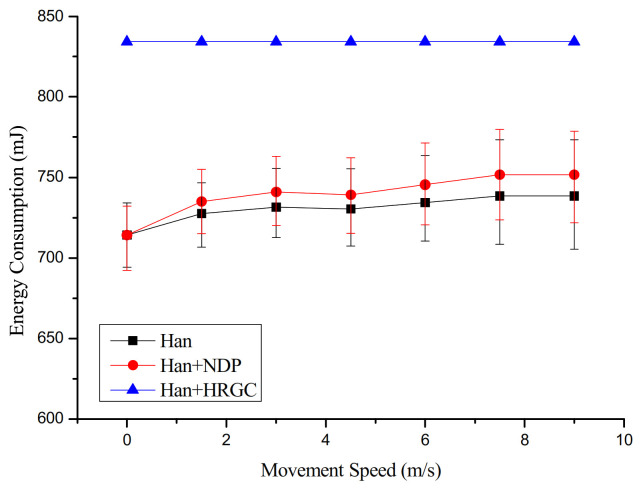
Energy consumption vs. movement speed of mobile devices.

Figure 8 shows the simulation result of the energy consumption versus the movement speed of mobile device. The Han algorithm shows the lowest energy consumption than other algorithm combination. As the original Han algorithm detects network changes according to the WirelessHART standard, the number of control messages used to detect network changes and reflect the update is the lowest. Therefore, the Han algorithm with NDP, which detects network changes faster than the existing WirelessHART standard, exploits more control messages than the original Han algorithm, and it consumes energy relatively high. In the case of Han algorithm with HRGC, it does not require the control message to detect network changes and reflect the update. However, in the packet transmission process, unnecessary waste occurs. For example, in Figure 4a, when the gateway transmits the broadcast message to all device in the network, the mobile device receives packet through the static device around itself. However, the packet also would be transmitted to the location where the mobile device does not actually exist. For example, even if the mobile device is located near S5, other static devices should try to transmit the broadcast message to mobile devices because they do not know if the mobile device is around them. Thus, the energy consumption of Han algorithm with HRGC is slightly higher than other algorithm combination.

**Figure 9:**
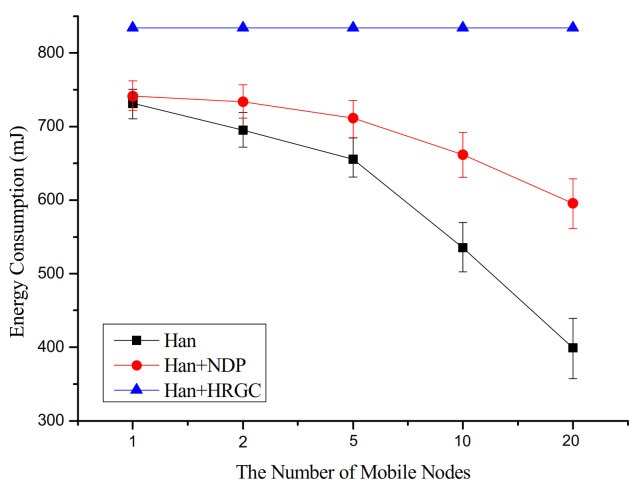
Energy consumption vs. the number of mobile devices.

Figure 9 shows the simulation result of the energy consumption versus the number of mobile devices. In the case of Han algorithm and Han algorithm with NDP, energy consumption is seen to decrease. It is because packet transmission failure due to graph failure has a greater impact on energy consumption than increasing control messages due to the increased number of mobile devices. That is, the increased number of mobile devices significantly reduces the packet transmission success rate, while also showing a degradation in energy consumption. However, in the case of Han algorithm with proposed method, the energy consumption consumes relatively higher energy than other combination, as some unnecessary energy waste increases proportionally to the number of devices.

## 5. Conclusions

In today’s industrial networks, many studies have been proposed to exploit wireless networks which supplement drawbacks of wired networks. However, the existing Industrial Wireless Networks (IWSNs), represented by WirelessHART, are primarily designed to alleviate for the shortcomings of wired networks such as wire installation and maintenance cost. Therefore, it is difficult to utilize the mobile devices widely used in today’s industrial environment. To overcome these problems, in this paper, we propose a HRGC for support mobile devices in IWSNs. The main idea of the HRGC is to construct a graph by separating static and mobile devices in order to prevent path failure and packet loss caused by the movement of mobile devices. First, exploiting the modified join message, the static and mobile device are classified, and a skeleton graph consisted of only the static device is constructed. As the routes based on the skeleton graph is not affected by the movement of mobile devices, it does not suffer problems such as packet loss. Then, by grafting the mobile devices into the skeleton graph, the HRGC establishes the routes for seamless communication of the mobile devices. Through this process, the mobile device could send/receive packets through the nearby static device in anywhere. The simulation results show that the packet delivery ratio is improved when the graph is constructed through the HRGC.

Although the HRGC ensures reliability and real-time in operation, it consumes relatively much energy in the initial process of grafting the mobile device into the skeleton graph. In order to reduce the energy consumption in the grafting process, we will perform further research to improve the HRGC through methods such as machine learning. In addition, there might be more mobile devices than static devices in various industrial environments. In the environment, HRGC could suffer from problems such as network disconnection (isolated of some static devices) in the process of constructing skeleton graphs. Therefore, we will also perform further research about networks with a large number of mobile devices.
